# Association of Alanine Aminotransferase Levels (ALT) with the Hepatic Insulin Resistance Index (HIRI): a cross-sectional study

**DOI:** 10.1186/1472-6823-12-16

**Published:** 2012-09-04

**Authors:** Miguel Ángel Gómez-Sámano, Daniel Cuevas-Ramos, Roopa Mehta, Hasan Brau-Figueroa, Clara Elena Meza-Arana, Alfonso Gulias-Herrero

**Affiliations:** 1 Department of Internal Medicine, Instituto Nacional de Ciencias Médicas y Nutrición “Salvador Zubirán”, Vasco de Quiroga # 15, Sección XVI Tlalpan, 14000 Mexico City, Mexico; 2 Department of Endocrinology and Metabolism, Instituto Nacional de Ciencias Médicas y Nutrición “Salvador Zubirán”, Mexico City, Mexico

**Keywords:** Alanine aminotransferase, ALT, Hepatic insulin resistance, HIRI

## Abstract

**Background:**

The association between serum alanine aminotransferase (ALT) levels and hepatic insulin resistance (IR) has been evaluated with the hyperinsulinemic-euglycemic clamp. However, there is no information about the association of ALT with the Hepatic Insulin Resistance Index (HIRI). The aim of this study was to evaluate the association between serum ALT levels and HIRI in subjects with differing degrees of impaired glucose metabolism.

**Methods:**

This cross-sectional study included subjects that had an indication for testing for type 2 diabetes mellitus (T2DM) with an oral glucose tolerance test (OGTT). Clinical and biochemical evaluations were carried out including serum ALT level quantification. HIRI was calculated for each participant. Correlation analyses and lineal regression models were used to evaluate the association between ALT levels and HIRI.

**Results:**

A total of 324 subjects (37.6% male) were included. The mean age was 40.4 ± 14.3 years and the mean body mass index (BMI) was 32.0 ± 7.3 kg/m^2^. Individuals were divided into 1 of 5 groups: without metabolic abnormalities (n = 113, 34.8%); with the metabolic syndrome (MetS, n = 179, 55.2%), impaired fasting glucose (IFG, n = 85, 26.2%); impaired glucose tolerance (IGT, n = 91, 28.0%), and T2DM (n = 23, 7.0%). The ALT (p < 0.001) and HOMA2-IR (p < 0.001) values progressively increased with HIRI quartiles, while ISI-Matsuda (p < 0.001) progressively decreased. After adjustment for sex, age, and BMI, we identified a significant correlation between HIRI and ALT in persons with the MetS (r = 0.22, p = 0.003), IFG (r = 0.33, p < 0.001), IGT (r = 0.37, p < 0.001), and T2DM (r = 0.72, p < 0.001). Lineal regression analysis adjusting for age, HDL-C, TG and waist circumference (WC) showed an independent association between ALT and HIRI in subjects with the MetS (beta = 0.07, p = 0.01), IFG (beta = 0.10, p = 0.02), IGT (beta = 0.09, p = 0.007), and T2DM (beta = 0.31, p = 0.003). This association was not identified in subjects without metabolic abnormalities.

**Conclusions:**

ALT levels are independently associated with HIRI in subjects with the MetS, IFG, IGT, and T2DM. The ALT value in these subjects may be an indirect parameter to evaluate hepatic IR.

## Background

The liver is an important organ for glucose metabolism; this includes glucose uptake, storage, and synthesis [1]. Studies have shown that nonalcoholic fatty liver disease (NAFLD) increases the risk for the metabolic syndrome (MetS) [2], type 2 diabetes mellitus (T2DM), and cardiovascular diseases [3-8]. Serum alanine aminotransferase (ALT) has been closely correlated with liver fat accumulation [9-11]. Therefore, this enzyme is commonly used as a biomarker of NAFLD [12]. In addition, clinical studies have associated serum ALT levels with insulin resistance (IR), the MetS and the development of T2DM [13-15].

In the fasting state, the rate of hepatic glucose production (HGP) is the main determinant of the fasting glucose concentration. Insulin normally inhibits HGP, however this suppression is not well achieved in subjects with hepatic insulin resistance. Therefore, elevated fasting insulin can be considered a surrogate marker of hepatic insulin resistance [16,17]. Accurate measurement of IR requires the use of techniques such as clamps that are costly, time-consuming, and invasive for use in large epidemiological or clinical studies. In this context, a number of simple indexes of IR have been proposed [18-23]. These indexes include HOMA-2, Matsuda-ISI, and QUICKI, among others.

A number of studies have reported a positive association between serum ALT levels and IR using indirect parameters such as the intravenous glucose tolerance test, (OR = 3.0 [95% CI 2.2-4.1]), and the homeostasis model assessment of insulin resistance [HOMA-IR, OR = 2.1 (1.5-2.9)] [3,14]. In the same way, a negative correlation has been reported between ALT and insulin sensitivity using the euglycemic hyperinsulinemic clamp (Clamp ISI, r = −0.44, p < 0.001) [15]. The association of ALT with hepatic insulin sensitivity measured by the euglycemic-hyperinsulinemic clamp (r = 0.21, p = 0.001) has been determined [13]. However, there is no information about the association of ALT with the insulin sensitivity index (ISI) of Matsuda [24] and HIRI [25]. The aim of the study was to evaluate whether an independent association exists between serum ALT levels and HIRI in subjects with differing degrees of impaired glucose metabolism.

## Subjects and methods

A total of 324 subjects fulfilled the selection criteria and were recruited for this cross-sectional study. The population consisted of subjects from the Internal Medicine and Endocrinology outpatient clinics of the Instituto Nacional de Ciencias Médicas y Nutrición “Salvador Zubirán” (INCMNSZ) that received attention between March 2007 and July 2010. We included individuals of both genders from 18 to 65 years-old, that had a 75 g oral glucose tolerance test (OGTT), and at least two risk factors for T2DM as stated by the American Diabetes Association (ADA) [26]. These include: physical inactivity, a first-degree relative with T2DM, high risk race/ethnicity, women with a history of delivering a baby weighing >4 kg or with a diagnosis of gestational diabetes mellitus during their pregnancy, arterial hypertension (blood pressure ≥140/90 mmHg or on therapy for hypertension), HDL cholesterol (HDL-C) level <35 mg/dL (0.90 mmol/L) and/or a triglyceride level >250 mg/dL (2.82 mmol/L), women with polycystic ovary syndrome, A1C ≥5.7%, IGT, or IFG on previous testing, other clinical conditions associated with insulin resistance (e.g., severe obesity, acanthosis nigricans), and a history of cardiovascular disease. HIRI and Matsuda indexes were calculated using the glucose and insulin values obtained every 30 minutes during the OGTT. We defined impaired fasting glucose (IFG, fasting glucose between 100 and 125 mg/dL), impaired glucose tolerance (IGT, 2 hr glucose value in the OGTT between 140 and 199 mg/dL), and type 2 diabetes (T2DM, fasting glucose level ≥ 126 mg/dL in two occasions, or glucose ≥ 200 mg/dL at the 2^nd^ hr of the OGTT) using ADA criteria [26]. The MetS was defined according to the U.S. 2004 National Cholesterol Education Program (NCEP) Adult Treatment Panel III guidelines [27] and modified as recommended in the latest American Heart Association/National Heart, Lung, and Blood Institute Scientific Statement for fasting glucose and waist circumference [28]. The MetS was defined as the presence of three or more of the following risk factors: 1) central obesity (waist circumference ≥80 cm in women and ≥ 90 cm in men) 2) hypertriglyceridemia (fasting triglycerides ≥150 mg/dl (≥1.69 mmol/l); 3) low HDL cholesterol (HDL cholesterol <50 mg/dl (<1.29 mmol/l) in women and <40 mg/dl (1.04 mmol/l) in men); 4) hyperglycemia (fasting glucose ≥100 mg/dl (≥5.6 mmol/l); and 5) arterial hypertension (sitting blood pressure of 130/85 mmHg or more, taken as a mean of two readings obtained after a rest of at least 10 min in this position). We excluded subjects with medications that could influence glucose and insulin values during the OGTT such as steroids, oral glucose lowering drugs (such as metformin, sulfonylureas, thiazolidinediones, acarbose, dipeptidyl peptidase-4 inhibitors, and glinides), subcutaneous insulin, and thyroid hormones. We also excluded subjects with other chronic diseases (such as HIV infection, hepatitis C infection (all subjects that received a blood transfusion before 1992 were tested for hepatitis C, other viral markers were not measured), systemic lupus erythematosus, rheumatoid arthritis, seizures, major depression, use of hepatotoxic drugs (i.e., acetaminophen, antibiotics, analgesics, chemotherapy among others) hospitalization in the past 6 months, active cancer or under treatment for cancer), and pregnant women. The population was divided into 5 groups according to the degree of impaired glucose metabolism: 1) without metabolic abnormalities defined as a patient without the MetS, IFG, IGT or T2DM; 2) with the MetS; 3) IFG; 4) IGT; and 5) T2DM. IR was estimated using HOMA2-IR calculator provided on the web page [18]; insulin sensitivity was estimated with the ISI Matsuda with the formula: 10000/√(Glucose 0’ x Insulin 0’) x (mean glucose x mean insulin) [24]. HIRI was evaluated using the formula described by Abdul-Ghani as follows: √(glucose 0 to 30 [AUC in mg/dl/hr] x insulin 0–30 [AUC in μU/ml/hr]) [25].

### Biochemical and anthropometric measurements

The central laboratory of the INCMNSZ performed all biochemical laboratory measurements. The measurements were carried out with commercially available standardized methods. Glucose, total cholesterol, HDL-cholesterol, and triglycerides were measured using the Synchron CX analyzer (Beckman Systems, Fullerton CA). Plasma insulin concentrations were estimated using a radioimmunoassay method (MEIA, Abbott Laboratories). ALT and aspartate aminotransferase (AST) were measured using an enzymatic method AU2700 Beckman Coulter (Fullerton, CA). Anthropometric measurements were carried out after participants removed their shoes and upper garments. Body weight was measured with a mechanical beam scale (Health o meter Inc, Bridgeview IL) with daily calibration. Body fat was measured with Quantum Desktop - BIA Analyzer by RJL systems. All subjects were instructed to stand in the center of the scale during weight assessment. Height was obtained using the floor scale’s stadiometer. Height was measured to the nearest 0.5 cm. WC was measured to the nearest 0.1 cm at the level of the greatest frontal extension of the abdomen between the bottom of the rib cage and the top of the iliac crest. BMI was calculated using weight (kg) divided by height squared (m^2^). Sitting blood pressure was measured after a rest of at least 10 min in this position.

### Ethics statement

The subanalyses presented in this study are from a protocol approved by our Human Biomedical Research Institutional Committee (REF 1650). Written informed consent was obtained from all subjects. All clinical investigation was conducted according to the principles expressed in the Declaration of Helsinki.

### Statistical analyses

Normally distributed data as assessed by the Kolmogorov-Smirnov test were expressed as mean and standard deviation (±SD) whereas variables with a skewed distribution were reported as median (interquartil range). The Pearson chi square test, Student’s unpaired *t*-test (normally distributed data), or Mann–Whitney *U* test (skewed distribution) was used as appropriate for comparisons between the sexes and for comparisons between subjects without metabolic abnormalities and subjects with differing degrees of impaired glucose metabolism. We divided the population in terms of HIRI quartiles: 0 to 28.2, 28.2 to 35.7, 35.7 to 43.2 and ≥ 43.2. Then, we compared these quartiles with the ALT and AST levels, peripheral and hepatic IR indexes, and the clinical and biochemical variables. One-way ANOVA (normally distributed data) or Kruskall–Wallis test (skewed distribution) was used for the comparisons between quartiles of the HIRI. Correlation coefficients between HIRI (adjusted for sex, age and BMI) and the clinical and biochemical parameters were evaluated with partial correlation analysis in each of the groups. We made six lineal regression models, one for each of the impaired glucose metabolism groups, to identify independent factors associated with HIRI. The variables selected to enter the regression analyses were those that correlated significantly with the HIRI. All reported p values are based on two-sided tests considering ≤0.05 as significant. All analyses were performed with SPSS 17.0 (Chicago, IL).

## Results

The characteristics of the study population, stratified by gender and the degree of impaired glucose metabolism are shown in Table 1. A total of 324 subjects (37.6% male) were included. The mean age was 40.4 ± 14.3 years with a BMI of 32.0 ± 7.3 kg/m^2^. The median (interquartil range) ALT concentration was 26.0 IU/L (20.0-41.0) with a HOMA2-IR, ISI Matsuda, and HIRI of 1.4 (0.9-2.0), 3.5 (2.3-5.5), and 36.9 ± 12.1, respectively. A total of 113 subjects (34.8%) without metabolic abnormalities, 179 subjects (55.2%) with the MetS, 85 subjects (26.2%) with IFG, 91 subjects (28.0%) with IGT, and 23 subjects (7.0%) with T2DM were evaluated. As is summarized in Table 1, significant differences were identified in ALT, HDL-C, and body fat percentage between males and females in each group.

**Table 1:** Clinical and biochemical characteristics of the subjects (n = 324)

**Variable**	**All subjects *(n = 324)***	**Without metabolic abnormalities *(n = 113)***	**Metabolic syndrome *(n = 179)***	**IFG *(n = 85)***	**IGT *(n = 91)***	**Type 2 DM *(n = 23)***
Age (year)	40.4 ± 14.3	36.4 ± 13.5	42.1 ± 14.3^a^	47.3 ± 13.9^b^	46.4 ± 14.7^c^	48.8 ± 12.7^d^
Males (n, %)	122 (37.6)	38 (33.6)	70 (57.4)	34 (27.9)	39 (32.0)	10 (8.2)
Weight (kg)	84.8 ± 22.2	80.1 ± 18.8	89.1 ± 20.8^a^	86.2 ± 21.6^b^	86.0 ± 22.1^c^	86.3 ± 17.9
BMI (kg/m^2^)	32.0 ± 7.3	29.9 ± 6.6	33.9 ± 8.3^a^	33.2 ± 9.3^b^	33.4 ± 9.0^c^	32.8 ± 4.7
WC (cm)	103.9 ± 16.7	98.4 ± 16.5	108.1 ± 15.5^a^	105.9 ± 16.1^b^	107.3 ± 16.3^c^	107.0 ± 13.9^d^
*M*	105.2 ± 14.8	96.8 ± 15.3	110.1 ± 12.9^a^	108.6 ± 13.5^b^	110.2 ± 13.2^c^	110.9 ± 7.5^d^
*F*	103.1 ± 17.7	99.2 ± 17.2	106.8 ± 16.9^a^	104.1 ± 17.5	105.1 ± 18.1	104.0 ± 17.0
p	0.26	0.48	0.16	0.20	0.14	0.24
SBP (mmHg)	118.6 ± 15.5	111.7 ± 12.4	123.2 ± 16.3^a^	124.3 ± 16.0^b^	124.2 ± 16.0^c^	131.3 ± 17.0^d^
DBP (mmHg)	77.7 ± 10.0	74.1 ± 9.8	80.1 ± 9.7^a^	79.3 ± 8.7^b^	79.9 ± 8.8^c^	81.6 ± 8.7^d^
ALT (IU/L)	26.0 (20.0-41.0)	22 (17.0-31.0)	27.0 (22.0-51.0)^a^	29.0 (22.0-60.0)^b^	31.0 (23.0-49.0)^c^	30.0 (24.0-61.0)^d^
*M*	33.0 (23.7-62.7)	29.0 (22.0-38.5)	40.0 (26.0-80.0)^a^	39.5 (26.0-79.2)^b^	33.0 (24.0-79.0)	34.5 (28.0-65.5)
*F*	23.0 (19.0-32.0)	20.0 (16–28)	25.0 (20.0-34.0)^a^	25.0 (21.0-38.0)^b^	29.0 (23.0-44.0)^c^	27.0 (23.0-55.0)
p	<0.001	0.001	<0.001	0.003	0.13	0.20
AST (IU/L)	25.0 (21.0-35.0)	23.0 (19.0-29.0)	26.0 (22.0-38.-0)	27.0 (22.0-41.5)	29.0 (23.0-42.0)	27.0 (23.0-45.0)
*M*	28.5 (22.0-41.2)	26.5 (21.0-34.5)	30.5 (23.0-48.2)	31.5 (25.0-47.2)	31.0 (23.0-45.0)	29.5 (23.7-45.7)
*F*	23.0 (20.0-3.5)	21.0 (180–27.2)	25.0 (21.0-32.0)	26.0 (22.0-37.0)	29.0 (22.2-37.5)	26.0 (22.0-50.5)
p	<0.001	0.005	0.001	0.074	0.455	0.832
TC (mg/dL)	189.0 ± 36.3	178.4 ± 36.5	195.9 ± 34.5^a^	197.0 ± 33.1^b^	191.4 ± 38.9^c^	190.5 ± 39.2
Triglycerides (mg/dL)	147.0 (108.7-201.5)	107.0 (75.7-131.0)	174.5 (149.7-245.2)^a^	163.0 (123.0-210.0)^b^	167.0 (121.0-236.5)^c^	170.5 (127.2-211.2)^d^
HDL-C (mg/dL)	37.7 ± 10.9	41.6 ± 12.6	34.7 ± 8.2^a^	37.1 ± 10.7^b^	35.2 ± 9.3^c^	30.6 ± 9.5^d^
*M*	32.5 ± 8.9	36.5 ± 10.5	30.9 ± 7.0^a^	32.1 ± 8.0	31.1 ± 8.1^c^	25.6 ± 4.4^d^
*F*	39.5 ± 11.0	44.4 ± 12.9	37.3 ± 8.0^a^	40.7 ± 11.0	38.1 ± 9.0^c^	35.6 ± 10.8^d^
p	<0.001	0.003	<0.001	<0.001	0.001	0.01
Body fat (%)	38.1 ± 9.1	37.1 ± 9.5	39.2 ± 8.8^a^	38.2 ± 8.9	38.3 ± 8.8	38.8 ± 8.1
*M*	30.9 ± 7.2	28.6 ± 8.8	32.4 ± 5.9^a^	31.0 ± 6.3	31.8 ± 6.4	32.1 ± 4.6
*F*	42.4 ± 7.1	41.4 ± 6.5	43.6 ± 7.5^a^	43.0 ± 7.0	43.1 ± 7.2	44.0 ± 6.1
p	<0.001	<0.001	<0.001	<0.001	<0.001	<0.001
FG (mg/dL)	93.7 ± 13.6	85.7 ± 8.1	98.2 ± 14.8^a^	110.3 ± 13.0^b^	104.8 ± 16.4^c^	123.0 ± 19.2^d^
Fasting insulin (μlU/mL)	10.8 (6.9-15.4)	7.3 (5.2-11.6)	12.8 (8.1-17.9)^a^	13.7 (9.2-18.6)^b^	13.4 (8.5-19.9)^c^	14.8 (7.0-21.6)^d^
HOMA-2IR	1.4 (0.9-2.0)	1.0 (0.7-1.5)	1.7 (1.1-2.4)^a^	1.8 (1.2-2.4)^b^	1.8 (1.1-2.6)^c^	2.1 (0.9-3.0)^d^
ISI Matsuda	3.5 (2.3-5.5)	5.5 (3.9-8.5)	2.8 (1.9-4.4)^a^	2.5 (1.5-3.4)^b^	2.5 (1.5-3.6)^c^	2.1 (1.4-3.6)^d^
HIRI	36.9 ± 12.1	33.1 ± 12.0	39.4 ± 11.9^a^	38.6 ± 12.4^b^	37.6 ± 10.3^c^	36.3 ± 10.9

Data are expressed as mean ± SD (comparisons made with Student unpaired t-test), median [(interquartil range), comparisons made with Mann–Whitney U test], or frequency [(%) comparisons made with the Pearson chi square] BMI = body mass index, WC = waist circumference, SBP = systolic blood pressure, DBP = diastolic blood pressure, ALT = alanine aminotransferase, AST = aspartate aminotransferase, TC = total cholesterol, HDL-C = high density lipoprotein cholesterol, FG = fasting glucose, HOMA2-IR = Homeostasis Model Assessment Insulin Resistance 2, ISI = Insulin Sensitivity Index, and HIRI = Hepatic Insulin Resistance Index.

a = p < 0.05 between subjects without metabolic abnormalities and MetS.

b = p < 0.05 between subjects without metabolic abnormalities and IFG.

c = p < 0.05 between subjects without metabolic abnormalities and IGT.

d = p < 0.05 between subjects without metabolic abnormalities and T2DM.

The total number of subjects is different of 324, because, 40% (n = 73) of subjects with MetS have IFG, 36% (n = 66) of subjects with MetS have IGT, and 11% of subjects (n = 20) of subjects with MetS have DM2.

**Figure 1:**
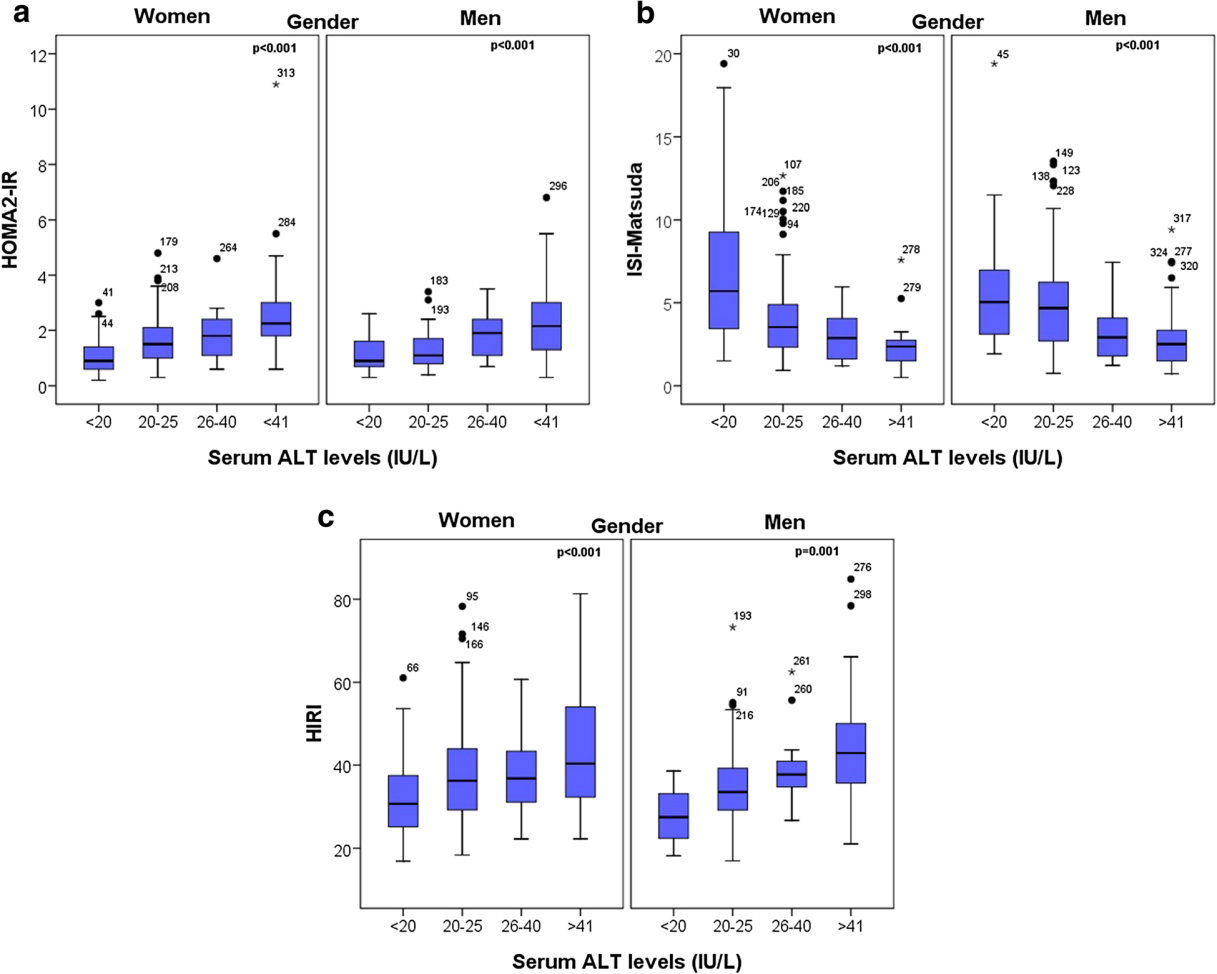
**Comparison of HOMA2-IR, ISI Matsuda and HIRI with serum ALT levels.a**. HOMA2-IR (n = 324, p < 0.001), and **c**. HIRI (n = 324, p < 0.001) significantly increased within ALT levels, whereas **b**. ISI Matsuda (n = 324, p < 0.001), significantly decreased within ALT levels, in both men and women. HOMA2-IR = Homeostasis Model Assessment for Insulin Resistance, ISI = Insulin Sensitivity Index, HIRI = Hepatic Insulin Resistance Index.

The baseline characteristics stratified by HIRI levels are shown in Table 2. In terms of demographic and laboratory data, a progressive and significant increment in weight (p < 0.001), BMI (p < 0.001), WC (p < 0.001), ALT (p < 0.001), body fat percentage (p < 0.001), insulin (p < 0.001), HOMA2-IR (p < 0.001) and HIRI (p < 0.001) were observed. In addition, HDL-C (p < 0.001) and ISI-Matsuda (p < 0.001) progressively decreased among HIRI quartiles. Interestingly, a higher level of HIRI and HOMA2-IR and a lower level of ISI-Matsuda index were identified among quartiles of ALT (Figure 1).

**Table 2:** Characteristics of the subjects studied stratified by quartiles of HIRI (n = 324)

**Variables**	**HIRI value (quartiles)**				
	**<28.2 *(n = 79)***	**28.2-35.7 *(n = 82)***	**35.7-43.2 *(n = 82)***	**≥43.2 *(n = 81)***	***p value***
Age (years)	41.3 ± 15.5	43.5 ± 14.7	40.0 ± 12.7	36.9 ± 13.9	0.03
Sex male (n, %)	28 (35.4)	29 (35.3)	35 (42.6)	30 (37.0)	0.74
Weight (kg)	75.4 ± 15.3	80.3 ± 18.4	90.8 ± 20.0	92.4 ± 21.8	<0.001
BMI (kg/m2)	28.5 ± 6.2	30.5 ± 6.4	34.5 ± 8.9	34.8 ± 7.4	<0.001
WC (cm)	95.1 ± 15.1	101.3 ± 15.0	108.8 ± 15.2	110.1 ± 17.0	<0.001
*Male*	96.0 ± 10.9	103.2 ± 12.6	108.4 ± 14.7	111.9 ± 16.0	<0.001
*Female*	94.6 ± 17.0	100.3 ± 16.2	109.0 ± 15.7	109.0 ± 17.7	<0.001
SBP (mmHg)	113.9 ± 14.1	121.0 ± 16.8	118.2 ± 14.4	117.4 ± 12.8	0.007
DBP (mmHg)	74.1 ± 10.9	77.6 ± 8.0	79.7 ± 9.7	77.4 ± 10.2	0.001
ALT (IU/L)	21.0 (15.0-29.0)	24.0 (19.7-34.0)	27.0 (21.0-46.0)	32.0 (24.0-68.0)	<0.001
*Male*	24.5 (15.5-40.2)	28.0 (23.0-36.0)	39.0 (26.0-61.0)	63.5 (31.0-93.0)	<0.001
*Female*	20.0 (15.0-26.0)	23.0 (19.0-30.0)	24.0 (20.0-37.0)	27.0 (22.0-40.0)	<0.001
AST (IU/L)	22.0 (19.0-29.0)	25.0 (21.0-31.0)	26.0 (21.0-37.0)	27.0 (22.5-45.5)	0.002
*Male*	24.5 (20.0-35.2)	28.0 (22.0-36.0)	29.0 (24.0-39.0)	38.5 (24.0-55.0)	0.026
*Female*	22.0 (19.0-27.0)	24.0 (20.5-29.0)	23.5 (19.0-34.2)	25.0 (21.0-36.0)	0.143
Cholesterol (mg/dL)	187.4 ± 39.5	190.2 ± 37.3	186.9 ± 36.5	191.0 ± 32.1	0.89
Triglycerides (mg/dL)	122.0 (81.5-166.0)	140.0 (95.0-204.2)	151.0 (114.2-204.0)	198.0 (148.5-348.5)	<0.001
HDL-C (mg/dL)	40.3 ± 12.5	40.5 ± 11.2	34.9 ± 8.1	35.1 ± 10.1	<0.001
*Male*	32.8 ± 8.8	36.8 ± 11.5	36.1 ± 8.1	29.6 ± 6.4	0.03
*Female*	44.6 ± 12.3	42.4 ± 10.7	39.8 ± 10.9	36.8 ± 8.9	0.001
Body fat (%)	35.0 ± 9.5	37.0 ± 8.7	39.8 ± 9.1	40.4 ± 8.0	<0.001
*Male*	27.0 ± 6.8	29.0 ± 5.6	33.0 ± 7.8	33.9 ± 6.4	<0.001
*Female*	39.4 ± 7.8	41.4 ± 6.8	45.0 ± 6.3	43.1 ± 7.4	<0.001
Glucose (mg/dL)	88.8 ± 12.9	94.2 ± 14.6	96.5 ± 12.7	95.1 ± 12.9	0.002
Insulin (μlU/mL)	6.5 (4.5-8.2)	8.2 (6.3-12.0)	13.1 (9.2-16.0)	17.6 (13.1-24.5)	<0.001
HOMA2-IR	0.8 (0.6-1.1)	1.1 (0.8-1.5)	1.7 (1.2-2.1)	2.3 (1.7-3.2)	<0.001
ISI Matsuda	6.9 (5.0-9.7)	4.6 (3.2-6.0)	2.9 (2.1-3.9)	2.0 (1.3-2.6)	<0.001
HIRI	23.4 ± 3.0	31.7 ± 2.1	39.0 ± 2.1	53.3 ± 9.5	<0.001

Data are expressed as mean ± SD, median (interquartil range), or frequency (%). Analyses were adjusted by gender. P values obtained of individual comparison using the Pearson chi square for frequency variables, one-way ANOVA (normally distributed variables) or Kruskall–Wallis (skewed distribution variables) test. BMI = body mass index, WC = waist circumference, SBP = systolic blood pressure, DBP = diastolic blood pressure, ALT = alanine aminotransferase, AST = aspartate aminotransferase, HDL-C = high density lipoprotein cholesterol, HOMA2-IR = Homeostasis Model Assessment Insulin Resistance 2, ISI = Insulin Sensitivity Index, and HIRI = Hepatic Insulin Resistance Index.

The partial correlations adjusted for gender, age and BMI between HIRI and the clinical and biochemical parameters are shown in Table 3. HIRI levels correlated significantly with ALT in all subjects with a metabolic diagnosis: MetS (r = 0.22, p = 0.003), IFG (r = 0.33, p < 0.001), IGT (r = 0.37, p < 0.001), and T2DM (r = 0.72, p < 0.001).

**Table 3:** Partial correlations of the HIRI with clinical and biochemical parameters

	**HIRI (adjusted for sex, age and BMI)**					
	**All subjects (n = 324)**	**Without metabolic abnormalities (n = 113)**	**Metabolic syndrome (n = 179)**	**IFG (n = 85)**	**IGT (n = 91)**	**T2DM (n = 23)**
**Variable**	***r***	***r***	***r***	***r***	***r***	***r***
Weight	0.07	−0.17	0.13	0.09	0.24^b^	0.13
WC	0.15^b^	0.04	0.16^b^	0.12	0.20	−0.31
SBP	0.09	0.05	0.05	−0.15	0.02	−0.13
DBP	0.08	0.09	0.01	−0.21	0.03	−0.02
ALT	0.20^a^	0.03	0.22^b^	0.33^a^	0.37^b^	0.72^a^
AST	0.22^a^	0.12	0.19^b^	0.26^b^	0.38^a^	0.51^b^
Cholesterol	0.01	−0.17	0.001	0.02	0.10	0.19
Triglycerides	0.19^a^	0.05	0.13	0.19	0.16	0.31
HDL-C	-0.15	-0.08	-0.08	-0.16	-0.05	0.21
Body fat	0.07	-0.03	0.10	0.01	0.19	0.09
Glucose	0.12^b^	0.16	0.03	0.01	0.09	0.29
Insulin	0.54^a^	0.63^a^	0.51^a^	0.56^a^	0.69^a^	0.81^a^
HOMA2-IR	0.53^a^	0.63^a^	0.51^a^	0.56^a^	0.69^a^	0.81^a^
ISI Matsuda	-0.59^a^	-0.60	-0.57^a^	0.61^a^	0.69^a^	-0.84^a^

P Values obtained of individual comparison using Spearman (normally distributed variable) or Pearson (skewed distribution variables) bivariate correlation tests: a = p < 0.001, b = p < 0.05. BMI = body mass index, WC = waist circumference, SBP = systolic blood pressure, DBP = diastolic blood pressure, ALT = alanine aminotransferase, AST = aspartate aminotransferase, HDL-C = high density lipoprotein cholesterol, HOMA2-IR = Homeostasis Model Assessment Insulin Resistance 2, ISI = Insulin Sensitivity Index, and HIRI = Hepatic Insulin Resistance Index. The total number of subjects is different from 324, because, 40% (n = 73) of subjects with MetS have IFG, 36% (n = 66) of subjects with MetS have IGT, and 11% of subjects (n = 20) of subjects with MetS have DM2.

Using six lineal regression models, adjusted for age, HDL-C, TG and WC we identified certain parameters with an independent association with HIRI levels (Table 4). The first model, that included all subjects, showed an independent association between ALT levels and HIRI (β = 0.07, t = 2.50, p = 0.013). In subjects without metabolic abnormalities we did not find any independent association between ALT levels and HIRI (β = 0.005, t = 0.08, p = 0.933). However, in models 3 to 6 that correspond to subjects with the MetS (model 3), IFG (model 4), IGT (model 5), and T2DM (model 6) we found an independent association between ALT and HIRI levels (p < 0.05 in all models, Table 4). Because of high collinearity between AST and ALT (r = 0.83, p < 0.001), we made different regression models utilizing AST instead of ALT. AST showed an independent association with HIRI (p < 0.05) in models: 1 (all subjects; beta = 0.08; t = 2.47; p = 0.014), model 3 (MetS; beta = 0.09, t = 2.47, p = 0.014), model 5 (IGT; beta = 0.11; t = 2.62; p = 0.011) and model 6 (T2DM; beta = 0.48; t = 3.35; p = 0.005). In individuals without metabolic abnormalities (model 2; beta = −0.04; t = −0.41; p = 0.681) and with IFG (model 4; beta = 0.08; t = 1.61; p = 0.11) we did not find any independent association between AST levels and HIRI ( Additional File 1: Table S1).

**Table 4:** Regression analysis of variables associated with the HIRI

**Variable**	**β**	**Standardized β**	**T**	**p value**
**Model 1 (all subjects, n=324)**				
Age	−0.11	−0.13	−1.86	0.064
ALT	0.07	0.17	2.50	0.013
HDL-C	−0.07	−0.05	−0.67	0.501
TG	0.009	0.114	1.60	0.111
WC	0.17	0.22	2.98	0.003
**Model 2 (Subjects without metabolic abnormalities, n=113)**				
Age	−0.05	−0.06	−0.57	0.569
ALT	0.005	0.009	0.08	0.933
HDL-C	−0.04	−0.05	−0.49	0.623
TG	0.013	0.065	0.61	0.541
WC	0.17	0.25	2.40	0.018
**Model 3 (Subjects with metabolic syndrome, n=179)**				
Age	−0.11	−0.13	−1.86	0.064
ALT	0.07	0.17	2.50	0.013
HDL-C	−0.07	−0.05	−0.67	0.501
TG	0.009	0.11	1.60	0.111
WC	0.17	0.22	2.98	0.003
**Model 4 (Subjects with impaired fasting glucose, n=85)**				
Age	−0.07	−0.08	−0.72	0.470
ALT	0.107	0.26	2.31	0.023
HDL-C	−0.05	−0.05	−0.39	0.697
TG	0.01	0.08	0.68	0.496
WC	0.08	0.11	0.97	0.334
**Model 5 (Subjects with impaired glucose tolerance, n=91)**				
Age	−0.17	−0.24	−2.41	0.018
ALT	0.09	0.28	2.78	0.007
HDL-C	0.02	0.02	0.201	0.841
TG	0.008	0.06	0.66	0.507
WC	0.14	0.22	2.18	0.032
**Model 6 (Subjects with type 2 diabetes, n=23)**				
Age	0.07	0.09	0.52	0.611
ALT	0.31	0.68	3.61	0.003
HDL-C	0.10	0.09	0.42	0.679
TG	0.01	0.15	0.75	0.463
WC	−0.09	−0.11	−0.48	0.636

Parameters of model 1: Constant: 23.6, F = 7.20; r^2^ = 0.17, p = <0.001.

Parameters of model 2: Constant: 17.72, F = 1.43; r^2^ = 0.07, p = 0.221.

Parameters of model 3: Constant: 23.6, F = 7.20; r^2^ = 0.17, p < 0.001.

Parameters of model 4: Constant: 27.7, F = 2.81; r^2^ = 0.16, p = 0.02.

Parameters of model 5: Constant: 23.0, F = 5.94; r^2^ = 0.28, p = 0.001.

Parameters of model 6: Constant: 21.0, F = 3.26; r^2^ = 0.53, p = 0.037.

ALT = alanine aminotransferase, HDL-C = high density lipoprotein cholesterol, TG = Triglycerides, WC = waist circumference, HIRI = Hepatic Insulin Resistance Index.

## Discussion

ALT levels have been associated with insulin resistance, the metabolic syndrome and the development of T2DM [13-15]. The aim of this study was to evaluate the association of serum ALT levels with surrogate markers of systemic and hepatic insulin resistance in subjects with and without impaired glucose metabolism. Since we excluded subjects on hepatotoxic drugs, or with liver disease or cirrhosis, this study suggests that ALT could be an indirect parameter reflecting the presence of hepatic insulin resistance in subjects with differing degrees of impaired glucose metabolism. We observed that in subjects without metabolic abnormalities, the association between ALT and HIRI was not statistically significant. However, in subjects with impaired glucose metabolism, or insulin resistance, ALT levels were an independent marker of hepatic insulin resistance as measured with HIRI. Measurement of ALT might be helpful as an early marker of metabolic abnormalities. We focused on ALT because this liver enzyme is more elevated in nonalcoholic steatohepatitis (NASH) than AST [29]. Also the linear regression models demonstrated that ALT levels showed association in all groups with impaired glucose metabolism. In contrast, AST was not statistically associated in the IFG group.

Initially, we identified several factors associated with a lineal increment in HIRI levels. We found that weight, BMI, WC, ALT, body fat percentage, insulin, and HOMA2-IR, increased progressively with higher quartiles of HIRI. Moreover, HIRI correlated positively with WC, ALT, TG, fasting glucose and insulin, HOMA2-IR; and negatively with HDL-c and ISI Matsuda. Furthermore, ALT was higher in men than women, an association that has previously been reported [30-32]. Only two previous studies have evaluated the association between ALT and hepatic IR; both of which used the euglycemic-hyperinsulinemic clamp [13,32]. Vozarova and cols [13] reported that higher ALT concentrations are associated with obesity, whole-body- and hepatic-IR. Furthermore, in a prospective observation, ALT levels were associated with a decline in hepatic insulin sensitivity and the development of type 2 diabetes. In our study, we used a novel hepatic IR index derived from the OGTT that can easily be applied in clinical practice. We found that HIRI correlated positively with serum ALT levels (Table 3). The significant association of HIRI with ALT confirms that an increment in this enzyme could be related to undiagnosed hepatic insulin resistance. Hepatic lipotoxicity caused by an oversupply of free fatty acids to the liver results in excess hepatic triglyceride synthesis and an intracellular accumulation of toxic lipid products that impair insulin signaling and activate inflammatory pathways [33]. The adaptation to this metabolic stress involves hepatic IR, dyslipidemia, steatohepatitis with mitochondrial dysfunction, endoplasmic reticulum stress, release of reactive oxygen species, and ultimately, hepatocellular damage [34].

The liver damage related to insulin resistance progresses to cirrhosis in approximately 20% of subjects with non-alcoholic steatohepatitis [35]. Therefore, early identification of metabolic abnormalities could be useful for initiating treatment and reducing the progression or even reverting the problem. Accordingly, we propose predicting the HIRI level using a single ALT measurement. According to model 3 (subjects with MetS), for each 10 IU/L of increment in ALT, the HIRI would increase approximately 0.7 units. However, a higher increment in HIRI could be expected if the patient has more severe impaired glucose metabolism. In subjects with IFG that corresponds to subjects with greater hepatic glucose production and hepatic IR [25], and in those with IGT that corresponds to subjects with impaired systemic IR [25] (models 4 and 5) for every 10 unit increment in ALT, HIRI increased by 0.9 and 1.0 unit respectively. In subjects with T2DM (model 6), an increment of 3 units of HIRI could be expected for the same 10 IU/L increment in ALT. This progressive increment in ALT with worsening glucose metabolism, possibly reflects how far along the patient is in the natural history of T2DM. The degree of hepatic insulin resistance could be predicted using the formula *HIRI = constant + beta (ALT level)*. The value of the *constant* and *beta* could be obtained from Table 4, depending on the patient diagnosis. If the patient does not have a metabolic diagnosis yet, we suggest using the information presented in model 1. We cannot confirm the association between ALT and fatty liver disease with the current design, however our hypothesis is that individuals with higher plasma concentrations of ALT and/or higher levels of HIRI could have an underlying liver disease that warrants further investigation. In addition, the benefit of metformin treatment in improving HIRI was recently reported by our group [36]. Similarly, another report has shown that metformin reduces ALT levels [30]. Perhaps, subjects identified with high normal or elevated serum ALT levels, without any other known liver disease, could benefit from metformin treatment to reduce hepatic insulin resistance. However, this point warrants further investigation in prospective studies. It is important to mention that our results could be potentially biased since we included patients that had an indication for testing for type 2 diabetes mellitus. Nevertheless, in the 113 patients that had no metabolic abnormalities we did not find any association between ALT and HIRI (Table 3, r = 0.03, p = 0.744; Table 4, model 2, beta = 0.005, p = 0.221). These results suggest that the increase in ALT in our patients is related to the metabolic derangement.

This study has certain limitations. Firstly, the cross-sectional design of our study does not allow us to conclude causality. Our results do not show that correction of insulin resistance with medication, diet, or exercising will improve ALT levels. Secondly, we used surrogate markers of insulin resistance. The results presented here should be confirmed in the clinical setting and studies with a prospective design. We did not take into account the amount of alcohol intake of the subjects in this study. In addition, our patients were not evaluated with liver ultrasound or gamma-glutamyl transpeptidase (GGT) levels, the results of which would aid patient diagnosis. Finally, these results can only be applied to similar individuals. Since ALT levels may differ by ethnic groups [37], our results should be confirmed in different populations.

## Conclusions

In conclusion, we found that ALT levels are independently associated with HIRI in subjects with the MetS, IFG, IGT, and T2DM. The ALT value in these subjects may be an indirect parameter to evaluate hepatic IR.

## Competing interests

The authors report no conflict of interest. The authors alone are responsible for the content and writing of the paper.

## Authors’ contributions

MAGS: Data collection, concept/design, data analysis, drafting article, statistics, critical revision of article; AGH: Concept/design; DCR: Concept/design, drafting article, statistics, critical revision of the article, data analysis; HBF: Design, data analysis, drafting article, statistics; RM: critical revision of the article, data analysis; CEMA: Data collection. All authors read and approved the final manuscript.

## Pre-publication history

The pre-publication history for this paper can be accessed here:

http://www.biomedcentral.com/1472-6823/12/16/prepub

## Supplementary Material

Additional file 1 **Table S1.** Regression analysis of variables associated with the HIRI. Model 1 (all subjects, n=324). Click here for file
