# Supplementary material for: Association of Alanine Aminotransferase Levels (ALT) with the Hepatic Insulin Resistance Index (HIRI): a cross-sectional study
Source: BMC Endocr Disord. 2012 Sep 4;12:16. doi: 10.1186/1472-6823-12-16 (PMC3515498; doi:10.1186/1472-6823-12-16)
Supplement: Additional file 1 — Table S1. Regression analysis of variables associated with the HIRI. Model 1 (all subjects, n=324). [file 1472-6823-12-16-S1.docx]

**Supplementary table 1.** Regression analysis of variables associated with the HIRI.

Model 1 (all subjects, n=324)

| **Variable** | **β** | **Standardized β** | **T** | **p value** |
| --- | --- | --- | --- | --- |
| Age | -0.07 | -0.09 | -1.69 | 0.092 |
| AST | 0.08 | 0.13 | 2.47 | 0.014 |
| HDL-C | -0.09 | -0.08 | -1.48 | 0.138 |
| TG | 0.013 | 0.145 | 2.58 | 0.010 |
| WC | 0.20 | 0.27 | 5.01 | <0.001 |

Parameters of model: Constant: 17.8, F=12.5; r^2^=0.18, p=<0.001

Model 2 (Subjects without metabolic abnormalities, n=113)

| **Variable** | **β** | **Standardized β** | **T** | **p value** |
| --- | --- | --- | --- | --- |
| Age | -0.06 | -0.06 | -0.60 | 0.545 |
| AST | -0.04 | -0.04 | -0.41 | 0.681 |
| HDL-C | -0.04 | -0.05 | -0.48 | 0.632 |
| TG | 0.015 | 0.075 | 0.69 | 0.487 |
| WC | 0.18 | 0.26 | 2.54 | 0.013 |

Parameters of model: Constant: 18.08, F=1.47; r^2^=0.07, p=0.205

Model 3 (Subjects with metabolic syndrome, n=179)

| **Variable** | **β** | **Standardized β** | **T** | **p value** |
| --- | --- | --- | --- | --- |
| Age | -0.13 | -0.15 | -2.09 | 0.038 |
| AST | 0.09 | 0.17 | 2.47 | 0.014 |
| HDL-C | -0.08 | -0.05 | -0.74 | 0.460 |
| TG | 0.009 | 0.11 | 1.65 | 0.099 |
| WC | 0.18 | 0.24 | 3.28 | 0.001 |

Parameters of model: Constant: 22.4, F=7.17; r^2^=0.17, p<0.001

Model 4 (Subjects with impaired fasting glucose, n=85)

| **Variable** | **β** | **Standardized β** | **T** | **p value** |
| --- | --- | --- | --- | --- |
| Age | -0.10 | -0.12 | -1.07 | 0.285 |
| AST | 0.08 | 0.17 | 1.61 | 0.110 |
| HDL-C | -0.06 | -0.06 | -0.45 | 0.649 |
| TG | 0.01 | 0.11 | 0.95 | 0.342 |
| WC | 0.11 | 0.14 | 1.19 | 0.238 |

Parameters of model: Constant: 27.8, F=2.20; r^2^=0.13, p=0.06

Model 5 (Subjects with impaired glucose tolerance, n=91)

| **Variable** | **β** | **Standardized β** | **T** | **p value** |
| --- | --- | --- | --- | --- |
| Age | -0.19 | -0.27 | -2.75 | 0.007 |
| AST | 0.116 | 0.26 | 2.62 | 0.011 |
| HDL-C | 0.02 | 0.02 | 0.201 | 0.838 |
| TG | 0.010 | 0.09 | 0.86 | 0.391 |
| WC | 0.16 | 0.25 | 2.47 | 0.016 |

Parameters of model: Constant: 21.5, F=5.72; r^2^=0.27, p=0.001

Model 6 (Subjects with type 2 diabetes, n=23)

| **Variable** | **β** | **Standardized β** | **T** | **p value** |
| --- | --- | --- | --- | --- |
| Age | -0.09 | -0.10 | -0.56 | 0.583 |
| AST | 0.48 | 0.65 | 3.35 | 0.005 |
| HDL-C | 0.22 | 0.21 | 0.94 | 0.362 |
| TG | 0.03 | 0.26 | 1.25 | 0.230 |
| WC | -0.008 | -0.01 | -0.04 | 0.969 |

Parameters of model: Constant: 11.1, F=2.86; r^2^=0.50, p=0.055

AST= aspartate aminotransferase, HDL-C=high density lipoprotein cholesterol, TG= Triglycerides, WC=waist circumference, HIRI=Hepatic Insulin Resistance Index.
